# AMBIVALENT AGEISM IN THE WORKPLACE AND ITS IMPACT: EXPLORING PERCEPTIONS OF OLDER WORKERS

**DOI:** 10.1093/geroni/igad104.0202

**Published:** 2023-12-21

**Authors:** Martine Lagacé, Ezgi Tasyurek, Philippe Rodrigue-Rouleau, Caroline Bergeron, Melanie Levasseur

**Affiliations:** Université d’Ottawa, Ottawa, Ontario, Canada; University of Ottawa, Ottawa, Ontario, Canada; University of Ottawa, Ottawa, Ontario, Canada; University of Ottawa, Ottawa, Ontario, Canada; Université de Sherbrooke, Sherbrooke, Quebec, Canada

## Abstract

Several countries are currently facing significant labour shortages in different work sectors. One of the solutions being considered to deal with such shortages is the retention of older workers. However, to do so, ageist attitudes and discrimination in the workplace must be countered as well as their negative impacts on older workers’ well-being. While previous studies have focused on assessing the impact of hostile ageism in the workplace, less research has been conducted on ambivalent ageism (i.e., stereotypes of fragility and incompetence) in the workplace. This study examines if and to what extent older workers perceive to be the target of ambivalent ageism and how such perceptions impact their well-being, in terms of psychological disengagement, self-esteem, perceived employability as well as intentions to leave their organization. An online, bilingual (French / English) questionnaire was completed by 951 Canadian older workers aged 50 years or more. Preliminary data analysis suggests that ambivalent ageism is negatively associated with perceived employability and self-esteem and positively associated with psychological disengagement and intentions to leave. Further, stratified data analysis by age group suggests that workers aged 62 or older perceive less ambivalent ageism, are less disengaged and have significantly higher self-esteem than workers of younger age groups. Such findings call for the implementation of workplace policies that are age-based inclusive and that account for differential experiences of ageism in the workplace.

